# Transitions between Central and Peripheral Vision Create Spatial/Temporal Distortions: A Hypothesis Concerning the Perceived Break of the Curveball

**DOI:** 10.1371/journal.pone.0013296

**Published:** 2010-10-13

**Authors:** Arthur Shapiro, Zhong-Lin Lu, Chang-Bing Huang, Emily Knight, Robert Ennis

**Affiliations:** 1 Department of Psychology, American University, Washington, D. C., United States of America; 2 Department of Psychology, University of Southern California, Los Angeles, California, United States of America; 3 Mayo Clinic, Rochester, Minnesota, United States of America; 4 SUNY College of Optometry, New York, New York, United States of America; Kyushu University, Japan

## Abstract

**Background:**

The human visual system does not treat all parts of an image equally: the central segments of an image, which fall on the fovea, are processed with a higher resolution than the segments that fall in the visual periphery. Even though the differences between foveal and peripheral resolution are large, these differences do not usually disrupt our perception of seamless visual space. Here we examine a motion stimulus in which the shift from foveal to peripheral viewing creates a dramatic spatial/temporal discontinuity.

**Methodology/Principal Findings:**

The stimulus consists of a descending disk (global motion) with an internal moving grating (local motion). When observers view the disk centrally, they perceive both global and local motion (i.e., observers see the disk's vertical descent and the internal spinning). When observers view the disk peripherally, the internal portion appears stationary, and the disk appears to descend at an angle. The angle of perceived descent increases as the observer views the stimulus from further in the periphery. We examine the first- and second-order information content in the display with the use of a three-dimensional Fourier analysis and show how our results can be used to describe perceived spatial/temporal discontinuities in real-world situations.

**Conclusions/Significance:**

The perceived shift of the disk's direction in the periphery is consistent with a model in which foveal processing separates first- and second-order motion information while peripheral processing integrates first- and second-order motion information. We argue that the perceived distortion may influence real-world visual observations. To this end, we present a hypothesis and analysis of the perception of the curveball and rising fastball in the sport of baseball. The curveball is a physically measurable phenomenon: the imbalance of forces created by the ball's spin causes the ball to deviate from a straight line and to follow a smooth parabolic path. However, the curveball is also a perceptual puzzle because batters often report that the flight of the ball undergoes a dramatic and nearly discontinuous shift in position as the ball nears home plate. We suggest that the perception of a discontinuous shift in position results from differences between foveal and peripheral processing.

## Introduction

The process of visual perception begins when an image of the external world forms on the retina in the back of the eye. The human visual system does not treat all parts of the image equally; rather, a disproportionate amount of neural processes are dedicated to the central two degrees of the image. At the level of the retina, the central region (the fovea) has a higher density of photoreceptors and ganglion cells than does the periphery, and (unlike other mammalian foveae) appears disproportionately populated by midget retinal ganglion cells [1] that have a characteristic morphology unlike other regions of the retina (see [2]). The central overrepresentation compared to the visual periphery can also be found in the lateral geniculate nucleus (LGN) [3] and to an even greater extent in the primary visual cortex [4], [5], [6]. The anatomical projections from the primary visual cortex to other cortical areas differ dramatically depending on whether those projections originated in the central or peripheral regions of the cortex [7]. In addition, projections from non-visual extrastriate cortical areas to V1 seem to target the peripheral visual cortex but not the central visual cortex [8].

Given the anatomical and physiological differences between the central and peripheral visual systems, it should not be surprising that the central portions of the visual image are seen at a higher resolution than the segments that fall in the visual periphery (see, for instance, [3], [9]). What is surprising, though, is that the distortions produced by the processing variation between central and peripheral vision often go unnoticed: our perceptual world appears as a seamless visual space composed from different views of the high-resolution portions of the image (for instance, when we look at a large object, we don't perceive the object as blurry at the edges) [10]. Here we examine what happens to our perception of a seamless visual space when the central and peripheral visual systems produce fundamentally different interpretations of the stimulus array. We ask whether our perceptual system will be able to integrate the conflicting responses, or whether the conflicting responses will lead to noticed distortions in spatial position and motion direction. These questions seem even more pertinent following recent research that has demonstrated that the peripheral visual system is not simply a scaled version of the central visual system, but also seems to lack the central visual system's ability to integrate features [11], [12].

**Figure 1:**
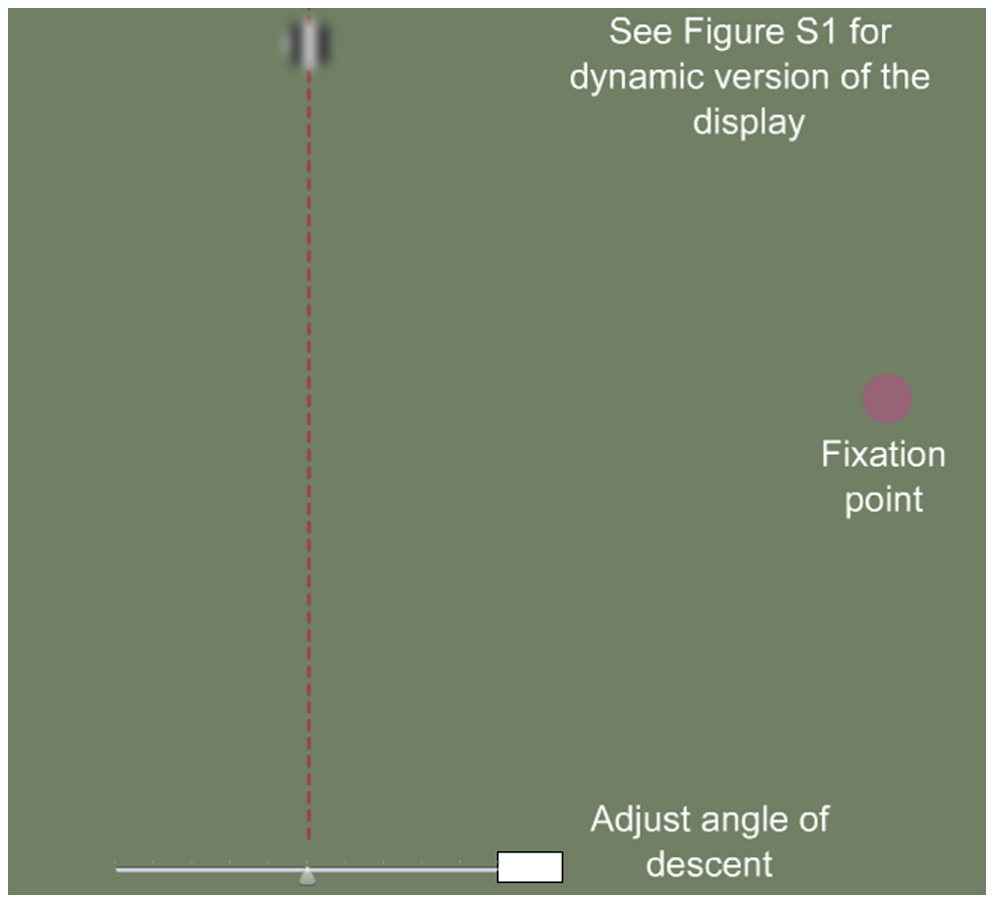
The curveball illusion (see Figure S1 for an interactive version of the illusion). A disk descends vertically from the top of the screen to the bottom. The inside of the disk consists of a sinusoidal grating that drifts horizontally from right to left. If the observer tracks the disk in central vision, the disk appears to descend vertically. If the observer tracks the disk in the periphery (i.e., if the observer looks to the right but attends to the motion of the disk), the disk appears to descend obliquely to the left. The effect can be made more dramatic if the observer shifts his/her gaze in the middle of the disk's descent, so that the object moves from the periphery to the fovea (or vice versa); this gaze shift creates a perceptual “jump” in which the direction of the disk snaps from an oblique descent to a vertical descent (periphery to fovea) or from a vertical descent to an oblique descent (fovea to periphery).

To investigate these issues, we developed interactive computer displays that accentuate the differences between central and peripheral perception (see [13], [14]). In this paper, we examine the curveball illusion (Figure 1; see Figure S1 for an interactive version), which juxtaposes two orthogonal motion signals: a global motion signal (i.e., the direction the disk travels across the computer screen); and a local motion signal (i.e., the internal spinning of that disk). In the curveball illusion, a disk descends vertically from the top center of the screen to the bottom center, while motion inside the disk is from right to left. If an observer tracks the disk foveally, the disk appears to descend vertically; however, if an observer shifts his/her gaze to the right so that the disk falls in the far visual periphery, the disk appears to drift to the left at an oblique angle (the perception of an oblique right shift can be created by left-to-right internal motion). The effect can be made more dramatic if the observer shifts his/her gaze in the middle of the disk's descent, so that the object moves from the periphery to the fovea (or vice versa). The gaze shift creates a perceptual “jump”: the direction of the disk snaps from an oblique descent to a vertical descent (periphery to fovea) or from a vertical descent to an oblique descent (fovea to periphery).

The effect demonstrates that the perceived position of an object can be altered when the angle of the object's internal motion differs from the angle of the object's global motion. Our experimental results are related to studies that indicate that the periphery integrates local and global motion [15], [16], [17], [18], [19]. Our version of the effect is most similar to the “infinite regress illusion” by Tse and Hsieh [20], which shows that the perceptual displacement can be particularly dramatic when the global motion is vertical while the internal grating moves horizontally. Our study adds to the motion literature and to the understanding of foveal and peripheral differences by drawing attention to the perceptual effect generated by the transition from central to peripheral vision (or vice versa) and to the effect's application to a specific (but not unique) real-world situation. While others have also noted the fundamental difference between interpretations of foveal and peripheral visual data in relation to eye movements [21], [22], we believe that our report is the first to call attention to the discrete shift in perceived direction that occurs when an observer shifts an image with global and local motion information from the fovea to the periphery, or vice versa; and the first to call attention to the ramifications of this gaze shift.

To measure the effects of viewing the curveball illusion in the periphery and the fovea, we developed a technique in which the disk falls at an oblique angle. The observer views the disk in the fovea and at different degrees in the periphery and reports when the disk appears to descend vertically. The angle at which the disk appeared to descend vertically was taken as the extent (or strength) of the illusion. We show that the central visual system separates these two motion signals, whereas the peripheral visual system reports a combination of the signals. The result is a compelling distortion in perceived direction that is amplified when the observer shifts the image from the central retina to the periphery, or vice versa.

Humans constantly shift objects from peripheral vision to central vision, and vice versa. We therefore propose that the perceived discontinuity in our experimental examples can be applied to real world situations. For instance, in the game of baseball, as a spinning ball travels from the pitcher's mound to home plate, the image of the ball is transferred from the batter's fovea to the batter's periphery (and vice versa). Batters often report that a curveball undergoes a discrete change in direction (the curveball's “break”) even though physical measurements indicate that the curveball curves gradually; batters also report that a fastball appears to rise when it is actually falling (the “rising fastball”). We therefore introduce a new hypothesis that these perceptual puzzles are due in part to the differing capabilities of the central and peripheral visual systems.

## Methods

### Ethics Statement

The experiments were conducted at the University of Southern California (USC). The USC Institutional Review Board approved the experimental protocol, and informed written consent was obtained from the observers.

### Observers

Two males and three females, 25 to 35 years of age, with normal or corrected-to-normal vision, including the third author, served as observers. Informed consent was obtained from all observers.

### Stimuli

The stimulus (similar to Figure S1) was generated in Adobe Flash CS3 and projected on a screen with a ViewSonic PJ 250 LCD projector. The background luminance was 54 cd/m^2^. At a viewing distance of 114 cm, the radius of the disk was 3.5 degrees. In each trial, the disk dropped for 2.4 seconds with a speed of 12.5 deg/sec. The disk contained two cycles of the grating. The internal grating moved either from left to right (direction = 0 degrees) or from right to left (direction = 180 degrees), with a velocity ranging from 6.7 to 20 deg/sec. The maximum velocity that can be achieved by our display is 20 deg/sec. In comparison, at 1500 rpm the internal rotation of a baseball is approximately 17 deg/sec when the ball is 20 feet away from the batter, but increases rapidly as the ball moves closer to the batter (e.g., 35 deg/sec at 10 feet and 70 deg/sec at 5 feet) [23]. These values were calculated by multiplying the ball's rotation speed (25 rotations/sec) by the visual angle subtended by the ball as it approaches the batter; the calculation can be described by the equation (*Rotation Speed* = 345.08/*Distance from batter*).

To aid judgment, a black vertical line (1cm*35cm) was drawn directly below where the disk starts falling. Two black diamonds with a side length of 4 cm were also provided as fixation targets, spaced horizontally 30 cm and 60 cm and vertically 30 cm (half the physical path) away from where the disk starts drifting, which corresponds to a horizontal retinal eccentricity of 15 and 30 degrees respectively.

### Design and Procedure

We estimated the magnitude of the illusion as a function of peripheral eccentricity by measuring the physical angle of descent that created the perception of vertical descent. The interactive demonstration program (Figure S1) contains a lever that permits a replication of the procedure. The experimenter adjusted the physical angle of descent, and the observer reported whether he/she perceived the disk to fall vertically. For example, the experimenter adjusted the global motion direction of the disk 20 degrees to the right if the observer reported, “No. The disk is moving to the left about 20 degrees.” The amount of adjustment became smaller as the observer reported that he/she saw the disk falling closer to vertical. The stimulus was on until the observer made a response, in response to which the experimenter changed the physical direction of the descending disk. The physical angle of deviation from the vertical at which the observer perceived a vertical descent was used to index the perceived illusion (in degrees). An observer's response was measured twice.

There were twenty-four different conditions: three eccentricities (0, 15 and 30 degrees), two directions for the internal grating (0 and 180 degrees), and four moving speeds (6.7, 10, 13.3 and 20 deg/sec). Each condition was repeated four times. Observers practiced 2 trials for each condition before data collection.

## Results

**Figure 2:**
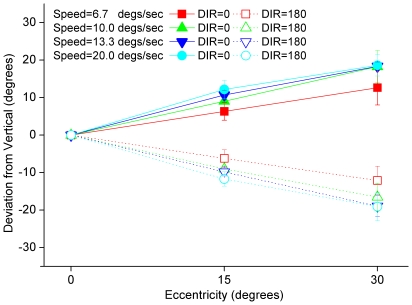
Perceived angle of deflection vs. eccentricity of test disk. The experimenter adjusted the physical angle of the disk's descent, and observers reported whether the disk appeared to descend vertically. The plot shows the angle reported as a function of viewing direction; each line represents a different speed for the internal grating. The perceived motion direction of the disk depends critically on how far the disk is from central vision.

The perceived motion direction of the spinning disk depends critically on how far the disk is from central vision (Figure 2). If the observer fixates on the disk, the disk appears to move down vertically with internal horizontal motion. Linear regression analysis (all r^2^>0.90) found that the perceived motion direction of the disk (for the average observer) deviates from vertical by about 0.41±0.04 (mean±s.d.), 0.58±0.04, 0.63±0.02, and 0.66±0.03× eccentricity (in degrees of visual angle) when the disk's internal motion is from right to left and the velocity is 6.7, 10.0, 13.3 and 20.0 deg/sec, respectively (−0.41±0.04, −0.58±0.04, −0.63±0.02, and −0.66±0.03× eccentricity when the disk's internal motion is from left to right). There is an almost significant but small dependence on the moving velocity (F(3,9) = 4.89, p = 0.03): the higher the moving speed, the bigger the deviation and, consequently, the stronger the illusion. For example, at an eccentricity of 30 deg, the disk appears to drop at a 20-degree angle from vertical when the velocity of internal motion is 20 deg/sec. Moreover, if the observer shifts his/her gaze in the middle of the disk's descent, so that the object moves from the periphery to the fovea or from the fovea to the periphery, a perceptual “break” occurs: from periphery to fovea, the disk snaps from a descent at an oblique angle to a vertical descent; from fovea to periphery, the disk snaps from a vertical descent to a descent at an oblique angle.

Foveal vision seems capable of separately representing and reporting the two orthogonal motion signals generated by the disk; peripheral vision, however, cannot represent the two motion signals separately. Rather, a single vector sum of the two orthogonal motion signals is perceived in peripheral vision. Because the relative strength of the two motion signals depends on eccentricity [24], [25], the perceived motion direction of the ball depends on eccentricity. Discrete changes in the perceived path of the ball arise when the image of the ball moves from central to foveal vision, or vice versa.

### Motion Energy in the curveball illusion

According to many (but not all) contemporary theories on motion perception, the global and local motion signals in the curveball illusion are perceived by the first- and second-order motion systems [26], [27], [28], [29], [30], [31], [32]. We can describe the motion energy used as an input to these models by performing a three-dimensional Fourier analysis (i.e., a Fourier analysis in x, y, t space) of the image cube, created by decomposing the movie in Figure S1 into a stack of still images (we extracted the still images from the movie with the aid of a Flash-Video converter, MacVide).

**Figure 3:**
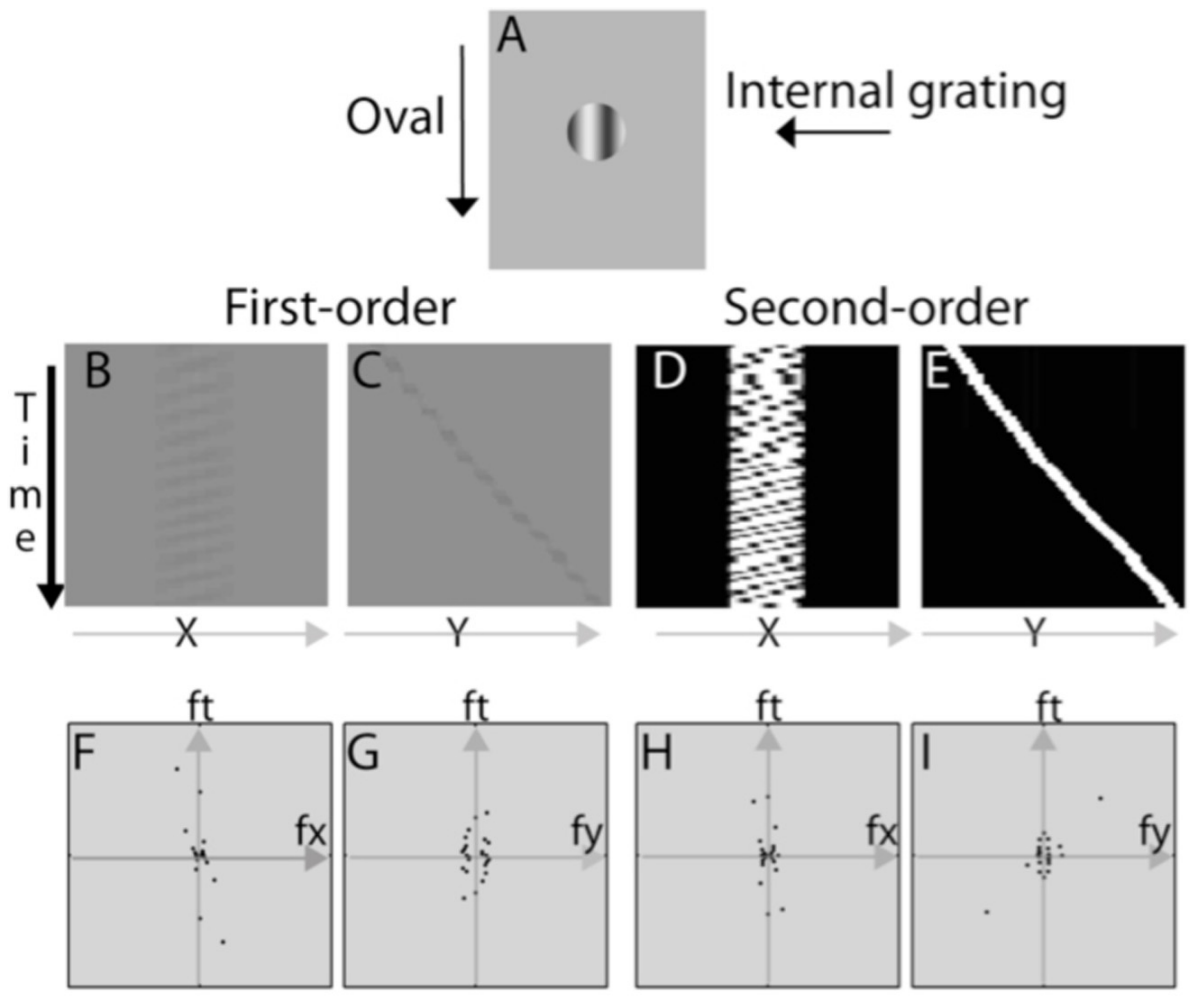
Motion analysis of the curveball illusion. A) A single frame of the curveball illusion. A disk falls from the top of the display to the bottom. Within the disk is an internal grating that moves from right to left. When viewed foveally, observers can separate the internal and the global motion signals, and the disk moves in a straight line; when viewed peripherally, the motion appears to move along an oblique trajectory. B&C) First-order motion plots in the x-t and y-t planes. D&E) Projections of DC-removed and rectified second-order curveball movie in the x-t and y-t planes. F-I) Fourier analysis of the first-order and second-order motion energy of the curveball movie in the fx-ft and fy-ft planes.

We can represent the motion of a spinning ball in a three-dimensional space by projecting the image cube on the x-t and y-t planes; these projections for the curveball illusion are shown in figure 3, panels B and C. To identify the second-order motion energy, we calculated the Michelson contrast of each point in each movie frame, removed the DC component in each frame by subtracting from the contrast images of each movie frame the mean x-y image of all the movie frames, and then applied a full-wave rectification to all of the resulting images [27], [29]. The projections of the second-order motion in the x-t and y-t planes are shown in figure 3, panels D and E.

The three-dimensional Fourier power spectrum was computed using Matlab 7.4 and then plotted on the fx-ft and fy-ft planes. Graphical representation of the first-order motion energy is shown in figure 3, panels F and G, and of the second-order motion energy, in panels H and I. The motion energy is represented as polar plots of the Fourier power summed over every 15 degrees in the fx-ft and fy-ft planes. Note that the different directions in the fx-ft and fy-ft planes represent different speeds in the horizontal and vertical directions. For any point in the fx-ft and fy-ft planes, the larger the slope of the line connecting the point to the origin, the faster the motion. In both the fx-ft and fy-ft planes, we define motion energy in quadrant *i*, 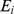, as the sum of Fourier power in that quadrant of Fourier space. The total motion energy, whose sign determines the direction of motion, is defined as
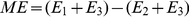
(1)In the fx-ft plane, positive motion energy signifies motion from the left to the right; negative motion energy signifies motion from the right to the left. In the fy-ft plane, positive motion energy signifies motion from the top to the bottom; negative motion energy signifies motion from the bottom to the top.

To take into account contrast-gain control in motion systems [33], a normalized measure of motion energy,
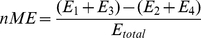
(2)was computed and used to estimate the presence or absence of horizontal and vertical motion in a display. 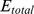 is the total Fourier energy in the four quadrants and on the axes.

For the first-order Fourier analysis: nMEx = −0.471 and nMEy = 0.031. The negative motion energy in the horizontal direction signifies right-to-left motion of the grating inside the disk. For the second-order motion analysis: nMEx = 0.416 and nMEy = −0.168. The negative motion energy in the horizontal direction signifies right-to-left motion, consistent with first-order analysis. So, both the first-order and second-order systems have significant motion energy in the right-to-left direction. The second-order system has significant motion energy in the top-to-bottom direction as well.

If one accepts a first- and second-order motion response to these stimuli, then the analysis indicates substantial first-order motion information in the horizontal direction, and significant second-order motion energy in both the horizontal and vertical directions. A system that responds to first-order motion would therefore record the ball as spinning but not falling, whereas as a system that responds to second-order motion would primarily record the ball falling. The perceived shift of the disk's direction in the periphery therefore is consistent with the integration of motion signals in both the first- and second-order motion systems.

### Relevance to the perceived path of the curveball

In the game of baseball, the pitcher throws a 2.9-inch-diameter ball in the direction of home plate, which is 60.5 feet away from the center of the pitcher's mound; the opponent (referred to as the batter) stands near home plate and attempts to hit the ball with a sturdy wooden bat. The pitcher makes the batter's task difficult by throwing the ball at different velocities and with different spins. One well-known type of pitch, the curveball, travels at about 75 mph with a 1500 rpm spin [23]. The curveball is a physically measurable phenomenon: the imbalance of forces created by the ball's spin causes the ball to deviate from a straight line and to follow a smooth parabolic path. However, the curveball is also a perceptual puzzle because batters often report that the flight of the ball undergoes a dramatic and nearly discontinuous shift in position as the ball nears home plate.

The most widely accepted theory on the perceived break of the curveball adequately explains why a batter might swing above or below the ball but does not account for the phenomenological appearance of a discontinuity in the curveball's path. This theory posits that batters estimate the ball's speed and direction from the first 0.4 seconds of the pitch, and a curveball leads the batter to overestimate the speed of the pitch [34]. If the batter's eyes are not fixated on the ball for the 0.15 seconds prior to the projected time to contact, the ball would take longer to reach the plate than the batter estimated and would curve more than the batter estimated. Here, we offer a theory that accounts for the perception of discontinuity: the curveball's break is a perceptual illusion caused by (1) the inability of peripheral vision to maintain separate representations of different motion signals, and (2) gaze shifts during the curveball's flight. The discontinuity is therefore a result of the change in the neural response at the transitional moment when the image of the ball – or a portion of the image – is transferred from the fovea to the periphery (or vice versa).

For the batter standing near home plate, the ball in the pitcher's hand has a visual angle of 0.23 degrees; the ball, when two feet away from home plate, has a visual angle of 6.89 degrees. Even if the batter could fixate on the center of the ball for the entirety of the ball's flight, the portion of the ball's image that falls outside the fovea increases over the course of the ball's flight. Batters, however, do not keep their eyes fixated on the ball for the entirety of its flight. Bahill and Baldwin (2004) propose two strategies for how batters track the ball. In the *optimal learning strategy*, batters follow “the first two-thirds of [the ball's] trajectory with smooth pursuit eye movements, make a saccadic eye movement to a predicted point of bat-ball collision, **continue to follow the ball with peripheral vision letting the ball catch up to the eye**, and finally, at the end of the ball's flight, resume smooth pursuit tracking with the images of the ball and bat on the fovea” [emphasis added]. In the *optimal hitting strategy*, batters “track the ball with smooth pursuit eye movements and fall behind in the last five feet.” Both strategies are consistent with the role we propose for gaze shift in the perceived break of the curveball: the image of the ball falls in the batter's fovea during some portions of the ball's flight toward home plate, in the periphery during other portions of the flight, and in the fovea and periphery as the ball approaches home plate. Therefore, the difference between central and peripheral vision is key to understanding the break of the curveball. Our experiment suggests that the visual periphery's representation of first- and second-order motion signals is relatively coarse compared to the fine separations maintained in foveal vision.

**Figure 4:**
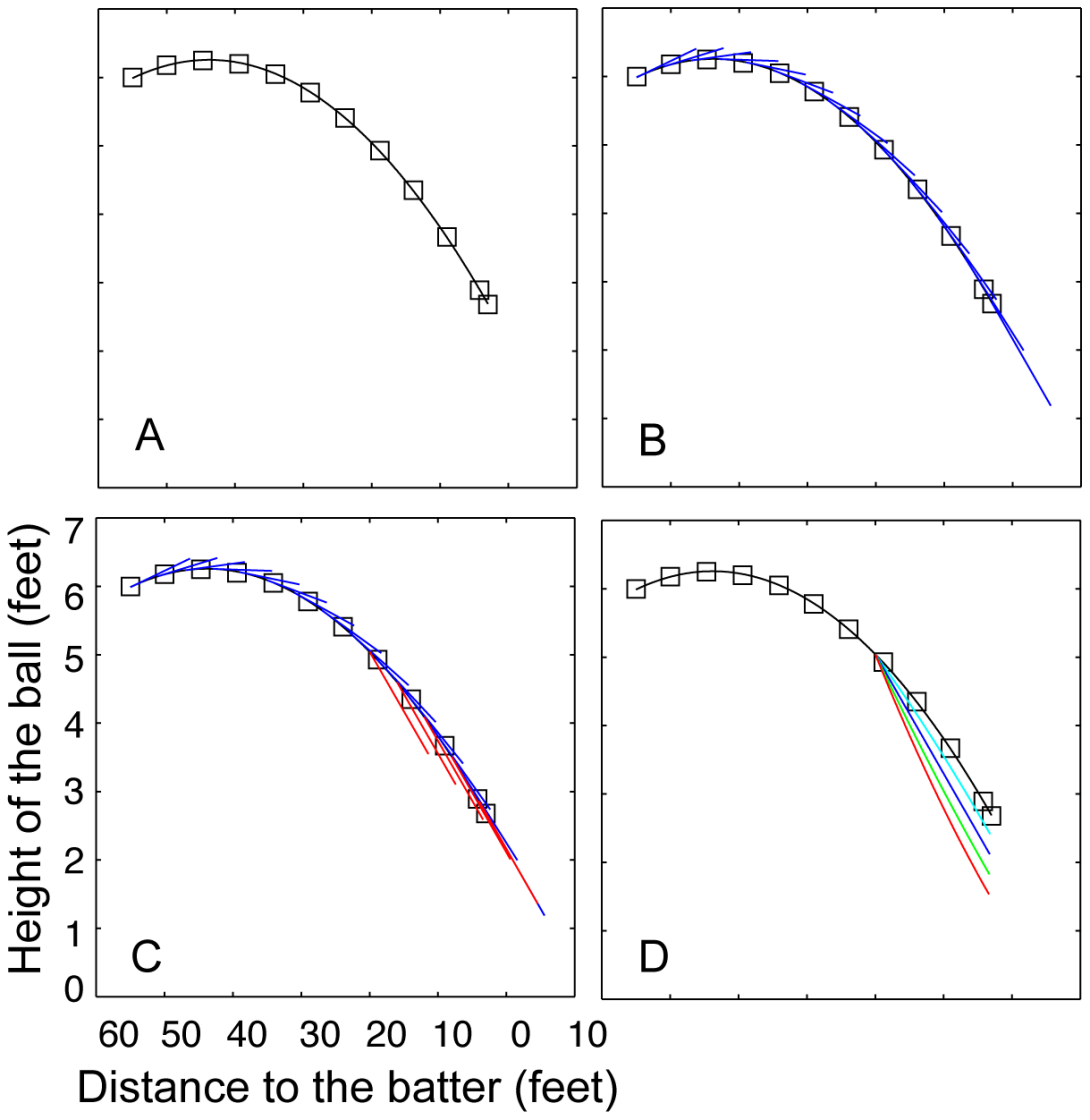
Experimental results applied to an actual trajectory of a curveball. A) The parabola fit to the curveball data tabulated in Bahill and Baldwin [34]. B) The line drawn at each point represents the physical velocity of the curveball at every moment of time. C) The deviation of the moment-by-moment perceived velocity of the ball (indicated by the red lines), assuming that the batter's gaze shifts to the expected point of bat/ball contact when the ball is 20 ft away from home plate (i.e., when the ball is 20 ft from home plate, the batter shifts his/her eyes so that the ball is at 10-degree eccentricity; the eccentricity decreases linearly when the ball reaches home plate). D) We used the perceived moment-by-moment velocity of the ball from part C to estimate the perceived trajectory of the ball, which is dependent on the initial eccentricity and when eye shift occurs. Each line indicates when the batter shifts his/her eyes from the ball toward home plate (i.e., the red line indicates that the observer shifts his/her eyes when the ball is 20 ft away; green line, 15 ft; dark blue line, 10 ft; light blue line, 5 ft). The longer the batter is able to maintain foveal fixation on the ball, the less the ball will be perceived to deviate from its parabolic path.

To demonstrate how our laboratory experiment is relevant to the break of the curveball in the field, we applied our experimental results to the actual vertical trajectory of a curveball (Figure 4A), as tabulated by Bahill and Baldwin (2004, table 10.2). First, we fit a parabola, 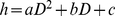 (where D is the distance between the ball and the batter), to the measured trajectory of a curveball (Figure 4A). The parameters were estimated using the “solver” function in Microsoft Excel; the values for parameters were a = 0.002, b = 43.808, and c = 6.257. The R^2^ value of the fitted function to the tabulated data equaled 1.00. We interpolated values of D that create equal interval spacing for the fitted equation (i.e., we found values of D such that ΔD^2^+Δh^2^ = Constant time). These values could be calculated interactively with the equation: D_n+1_ = sqrt (time_interval/(1+4*a* ^2^(D_n_−*b*)^2^)). We computed the physical velocity of the curveball at every moment of time (Figure 4B).

To compute the moment-by-moment perceived velocity of the ball, we added a 0.66×eccentricity (degrees) deviation to the physical velocity (Figure 4C); the calculation assumed that the batter's gaze shifts to the expected point of bat/ball contact when the ball is 20 feet away from home plate, and the batter at that moment sees the ball with a 10-degree eccentricity, and the eccentricity linearly reduces to 0 degrees as the ball travels the final 20 feet to home plate [34]. Lastly, we repeated the third step for eccentricities 5, 15, and 20 degrees, and computed the perceived moment-by-moment trajectory of the ball (Figure 3D).

The analysis indicates that if the batter shifts his/her gaze to fixate on the ball at any point on the perceived path (i.e., if the batter resumes viewing the ball foveally), a break of up to 1.25 ft will be perceived (depending on the initial eccentricity and when eye shift occurs). A similar analysis could be applied to the horizontal motion of the curveball or to the perceived rise of a fastball.

## Discussion

The hypothesis presented here connects the break of the curveball to a growing literature that demonstrates dramatic differences between central and peripheral vision. A longstanding question in vision science concerns the effects that anatomical and physiological differences in the fovea and periphery have on visual function. One prominent hypothesis is that vision in the periphery is primarily a spatially, temporally, and photometrically scaled version of vision in the fovea. Such a view is supported by findings that grating sensitivity and Vernier acuity measured in the periphery match measurements in the fovea scaled by a factor that accounts for the differing distribution of ganglion cells [35], [36]. However, other findings suggest that vision in the periphery cannot be fully explained by the scaling of foveal vision [11], [12]. Our results indicate that the peripheral visual system combines features that the foveal visual system can process separately [37], [38], [39].

Humans are consistently changing fixation, such that information processed in the periphery can be processed in the fovea, and vice versa. We have shown that motion shifts from the fovea to the periphery are consistent with a motion model in which the foveal visual system responds separately to first- and second-order motion information, and the peripheral system combines first- and second-order motion information. Motion shifts could result from a capacity limitation for segregating features in the periphery (as in models that account for visual crowding [11], [12]) or from different spatial and temporal weightings of first- and second-order motion information as the stimulus moves away from the fovea. There are, however, other motion models that could account for motion in these stimuli without recourse to first- and second-order motion processes. For instance, the multichannel gradient model [40], [41], [42] combines motion signals at different scales. It is likely that such a model could be adjusted to account for a shift in direction from fovea to the periphery; however, we note that optically blurring the stimulus does not create a dramatic change in the perceived direction of the ball. Therefore, it would seem that a gradient model would have to include additional parameters to account for the combination of motion signals at later stages of processing.

Lastly, we propose that this discrete shift in perceived direction is directly germane to the perceived flight of spinning balls. In the game of baseball, if a batter could track a curveball's entire flight, a good portion of the ball would be in his/her peripheral vision when the ball approaches home plate. Since batters cannot maintain foveal eye-tracking, the shift from central to peripheral vision (and vice versa) is more dramatic. We therefore contend that a model that emphasizes limited capacity (or different temporal and spatial weighting) in the periphery has all the properties necessary to contribute to the perceived break of the curveball, and that the shift from foveal to peripheral vision (and vice versa) underlies the batter's perception of a break.

A similar principle may also explain illusions associated with fastballs. Typical major league fastballs travel at 90 mph, with a 1200 rpm backspin. Fastballs descend on their way from the pitcher's mound to home plate, but batters often report the perception that fastballs rise [43]. The perception of a rise is consistent with how a batter's peripheral vision would perceive a ball with a backspin. There are two different types of fastballs, a two-seam and a four-seam; batters and pitchers report that the two balls appear to travel with different trajectories even though wind-tunnel analysis has shown no difference between the lifts produced by the different spins [44]. Bahill and Baldwin (2004) estimated that the spin of the two-seam fastball would be below the human flicker threshold, whereas the spin of the four-seam fastball would be above flicker threshold (assuming that the two-seam fastball spins so that the two seams cross the batter's field of view on each rotation of the ball, and the four-seam fastball spins so that the four seams cross the batter's field of view on each rotation). Research with realistic baseball simulators or field studies with eye-tracking equipment are necessary to further understand the perceived directions of these pitches.

The curveball illusion addresses a more general problem for perceptual theory. We have shown that a single distal stimulus (a spinning disk that moves vertically down the screen) produces a perception in the fovea that differs from the perception in the periphery. The human visual system must therefore integrate fundamentally different interpretations of the higher-order information available in the extended visual stimulus. Our results give emphasis to [10] prescient and cautionary comment that a “prudent” vision researcher “would worry somewhat about the matter [of the extended stimulus array] before either making practical prescriptions to designers of aircraft displays and landing strips, or building theoretical and philosophical structures on the assumption that such information from extended stimulus arrays is in fact the predominant basis of normal perception.” Humans constantly shift objects between central and peripheral vision and may encounter effects like the curveball's break regularly. Peripheral vision's inability to separate different visual signals may have far-reaching implications in understanding human visual perception and functional vision in daily life.

## Supporting Information

Figure S1 Interactive Curveball Illusion. A disk descends vertically from the top of the screen to the bottom. If the observer tracks the disk in the periphery (i.e., if the observer looks to the right but attends to the motion of the disk), the disk appears to descend obliquely. The lever allows the observer to adjust the angle of descent. Experiment 1 measured the physical angle of descent at which the observer perceived the disk to descend vertically when viewing the disk in the periphery.
(0.03 MB SWF)
